# Controversies in the diagnosis of chronic inflammatory demyelinating polyneuropathy

**DOI:** 10.1097/WCO.0000000000001414

**Published:** 2025-08-01

**Authors:** Pietro Emiliano Doneddu, Carla Fasano, Claudia Lozi, Gaia Marenna, Eduardo Nobile-Orazio

**Affiliations:** a Neuromuscular and Neuroimmunology Unit, IRCCS Humanitas Research Hospital, Rozzano; b Department of Biomedical Sciences, Humanitas University, Pieve Emanuele; c Humanitas University, Pieve Emanuele, Milan; d Department of Medical Biotechnology and Translational Medicine, Milan University, Milano, Italy

**Keywords:** antimyelin-associated glycoprotein neuropathy, axonal damage, chronic inflammatory demyelinating polyradiculoneuropathy, electrodiagnostic criteria, neurofilaments

## Abstract

**Purpose of review:**

Despite decades of clinical recognition, the diagnosis of chronic inflammatory demyelinating polyradiculoneuropathy (CIDP) remains fraught with uncertainty. This review examines major areas of ongoing controversy in the diagnostic evaluation of CIDP, focusing on recent changes to electrodiagnostic criteria, disease boundaries, and emerging concepts of axonal damage.

**Recent findings:**

Recent literature highlights three key areas of diagnostic uncertainty: the evolution and limitations of electrodiagnostic criteria; the diagnostic boundary between CIDP and antimyelin-associated glycoprotein (anti-MAG0 antibody neuropathy; and the recognition of CIDP cases that do not fulfil electrodiagnostic criteria, raising interest in axonal variants and the potential role of biomarkers such as neurofilaments. Across these domains, discrepancies between empirical evidence and expert-based guidelines persist, contributing to misdiagnosis and treatment variability.

**Summary:**

Current CIDP criteria, though improved, remain partly based on expert opinion rather than empirical validation. The clinical heterogeneity of CIDP and its overlap with mimicking disorders further complicate diagnosis. A broader, more flexible diagnostic framework − integrating electrophysiology, biomarkers, and treatment response − is essential to enhance diagnostic accuracy and guide therapy. Future research should focus on refining criteria to strengthen electrodiagnostic standards and better accommodate atypical and axonal presentations.

## INTRODUCTION

Chronic inflammatory demyelinating polyradiculoneuropathy (CIDP) is an immune-mediated disorder of the peripheral nervous system characterized by progressive or relapsing motor and sensory dysfunction [1]. First described several decades ago [2], CIDP has since been the focus of extensive clinical and research attention. Yet, despite its long-standing recognition, significant controversies persist − particularly regarding its diagnosis. These challenges stem from the clinical and electrophysiological heterogeneity of the disease, the overlap with other neuropathies, and evolving diagnostic criteria. This review will focus on three of the most debated controversies in CIDP, aiming to clarify current perspectives and highlight areas of ongoing uncertainty. 

**Box 1:**
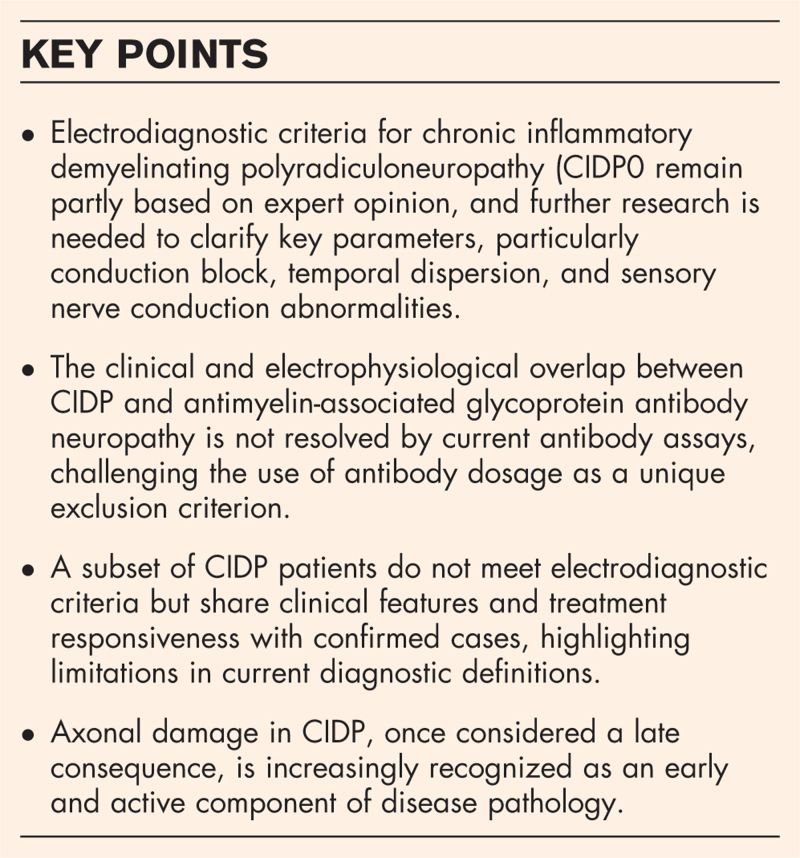
no caption available

## ELECTRODIAGNOSTIC CRITERIA FOR CHRONIC INFLAMMATORY DEMYELINATING POLYRADICULONEUROPATHY: BETWEEN PRACTICE AND EVIDENCE

The diagnosis of CIDP relies on clinical history and neurological examination in combination with nerve conduction studies and supportive investigations, including cerebrospinal fluid (CSF) analysis, neuroimaging, and nerve biopsy [3^▪^]. Among these, nerve conduction study remains the most essential tool for confirming the diagnosis, as it provides objective evidence of demyelination through predefined electrodiagnostic criteria [3^▪^]. These criteria have evolved considerably over time. Since the term CIDP was first introduced by Dyck and colleagues in 1975, more than 15 sets of diagnostic criteria have been proposed [3^▪^,^4^–^9^]. Early criteria were primarily developed for research purposes and prioritized diagnostic specificity, whereas more recent iterations have aimed to improve sensitivity. In a rare and disabling disease such as CIDP − where effective treatments are available − achieving high sensitivity is desirable to ensure that the maximum number of patients receive appropriate therapy.

However, a growing body of literature has highlighted concerns regarding diagnostic inaccuracy, particularly overdiagnosis, in both the United States and Europe [10,11]. In one misdiagnosed cohort, 44% of patients fulfilled the 2010 European Federation of Neurological Societies/Peripheral Nerve Society (EFNS/PNS) criteria, thus drawing attention to the specificity of current diagnostic standards [10]. In response, the 2021 European Academy of Neurology/Peripheral Nerve Society (EAN/PNS) criteria sought to improve specificity through further refinement [3^▪^].

Many of the modifications introduced in the latest criteria − and, to a large extent, also in prior versions − stem from expert opinion rather than robust empirical evidence, raising questions about their scientific validity. A key example is the definition of motor conduction block, which has undergone significant revisions over time, with no consensus on the optimal criteria [3^▪^,^4^–^9^,^12^–^17^]. The main issues include whether to assess the block by a reduction in compound muscle action potential (CMAP) area, amplitude, or both; the threshold for abnormality − which has ranged from 20% to 50%; whether temporal dispersion should be an exclusion criterion; and whether the threshold should be adjusted according to distal CMAP size [3^▪^,^4^–^9^,^12^–^17^,^18^].

An additional point of interest is the divergent definitions of conduction block in CIDP versus multifocal motor neuropathy (MMN), another chronic immune-mediated neuropathy. In CIDP, conduction block is defined based on amplitude reduction without temporal dispersion limits [3^▪^,^4^–^7^,^9^], whereas in MMN, it is based on area reduction with temporal dispersion used as an exclusion criterion [13,19]. This discrepancy may reflect the enduring influence of early MMN studies that emphasized conduction block in isolation, prompting the adoption of a conservative definition [20]. Specifically, some studies suggest that CMAP area is better than amplitude at distinguishing true conduction block from apparent block due to temporal dispersion [12–14,21].

In CIDP, temporal dispersion is itself a diagnostic feature, which obviates the need for overly restrictive criteria [3^▪^,^4^–^9^]. The 2010 EFNS/PNS criteria for CIDP defined definite conduction block as a ≥50% reduction in CMAP amplitude and probable block as a ≥30% reduction, provided the distal CMAP was at least 20% of the lower limit of normal [4]. In contrast, the 2021 EAN/PNS criteria adopted a unified threshold of ≥30% amplitude reduction, eliminated the distinction between probable and definite block, and excluded the tibial nerve due to its susceptibility to technical artefacts and entrapment [3^▪^]. Comparative studies indicate that these revisions improved specificity without compromising sensitivity, thereby supporting their adoption [22,23,24^▪^].

Temporal dispersion has also undergone repeated redefinition. The threshold has varied from 15% to 30% [3^▪^,^4^–^7^,^21^,^25^,^26^], and its measurement in the tibial nerve has been excluded [3^▪^]. Debate continues over whether temporal dispersion should be assessed based on the increase in duration of the negative peak or the total duration of the CMAP [21,27]. Multiphasic responses, which are common in CIDP, complicate measurement of the negative peak alone and may obscure true dispersion [21,27]. Nonetheless, the negative peak method remains preferred in recent criteria due to its perceived reproducibility; the gradual tapering of the CMAP's terminal component complicates the accurate identification of the end of the total duration [21]. However, this approach is supported by limited empirical data, and a large-scale study comparing methods in well characterized patients and controls is lacking. The 2021 EAN/PNS criteria retained the 30% threshold for temporal dispersion based on prolongation of the negative peak duration, while increasing the cutoff to 100% for the tibial nerve [3^▪^]. This change has been validated in recent studies, showing improved specificity with only a minimal sacrifice in sensitivity [22,23,24^▪^].

The role of sensory conduction abnormalities in the electrodiagnostic assessment of CIDP has similarly evolved − from being a marginal feature to becoming a core diagnostic criterion. Early literature emphasized the distinctive finding of reduced or absent sensory nerve action potentials (SNAPs) in the upper limbs, with relative sparing of the sural nerve [28–30]. This pattern contrasts with the length-dependent sensory loss typically seen in axonal neuropathies. Subsequent studies evaluated additional parameters such as sensory conduction velocity slowing, sensory conduction block, and increased SNAP duration [31,32]. However, these studies were often limited by small sample sizes and heterogeneous control groups, with some excluding diabetic patients. Despite these limitations, most investigations concluded that incorporating sensory nerve conduction abnormalities enhanced diagnostic sensitivity without significantly compromising specificity [29–32]. In particular, Tamura and colleagues, in the largest study to date including both CIDP and control subjects (with and without diabetes), reported a sensitivity and specificity of 32% and 95–96% respectively for an abnormal median but normal sural SNAP, and 18% and 94–96% respectively for an abnormal radial but normal sural SNAP [29]. Rajabally and Samarasekera, in a smaller cohort, also demonstrated high diagnostic value for sensory demyelinating features, including reduced conduction velocity and sensory conduction block [31]. The first formal recognition of sensory abnormalities came from the American Academy of Neurology (AAN) Ad Hoc Subcommittee criteria, which included a reduction in sensory conduction velocity below 80% of the lower limit of normal as a “supportive” finding for CIDP [7]. The 2010 EFNS/PNS criteria further endorsed sensory abnormalities as supportive features, specifically incorporating reduced sensory conduction velocity (<80% lower limit of normal [LLN], or <70% if SNAP amplitude <80% LLN) and preserved sural responses with abnormal median or radial SNAPs (excluding carpal tunnel syndrome) [4]. A major shift occurred with the 2021 EAN/PNS criteria, which elevated sensory abnormalities to a mandatory diagnostic requirement, specifying the presence of sensory abnormalities in at least two nerves [3^▪^]. However, two comparative studies have shown that this modification resulted in reduced sensitivity of the 2021 EAN/PNS criteria, with only minimal or no improvement in specificity, suggesting that while sensory parameters have diagnostic value, mandating their presence may exclude a subset of true CIDP cases [22,23,24^▪^].

## BOUNDARIES BETWEEN CHRONIC INFLAMMATORY DEMYELINATING POLYRADICULONEUROPATHY AND ANTIMYELIN-ASSOCIATED GLYCOPROTEIN ANTIBODY NEUROPATHY

Another debated aspect in the diagnosis of CIDP is its differentiation from antimyelin-associated glycoprotein (MAG) antibody neuropathy, a condition that shares several clinical and electrophysiological features with CIDP. In most cases, this distinction is straightforward. Unlike CIDP, anti-MAG antibody neuropathy typically presents as a slowly progressive, predominantly sensory, length-dependent polyneuropathy, often accompanied by tremor and ataxia [33^▪▪^]. Motor involvement is generally less prominent, appears later in the disease course, and rarely affects proximal limb segments [33^▪▪^]. The electrophysiological profile also differs, albeit with some overlap, showing greater involvement of distal nerve segments and a lower frequency of conduction blocks [33^▪▪^]. Furthermore, an immunoglobulin M (IgM) monoclonal gammopathy is nearly always present, and anti-MAG antibodies − considered the diagnostic hallmark − are invariably detectable [33^▪▪^]. Both the 2010 EFNS/PNS and the 2021 EAN/PNS diagnostic criteria for CIDP list anti-MAG antibody positivity as an exclusionary factor [3^▪^,^4^].

Nonetheless, in a minority of cases, the clinical distinction becomes less clear-cut. The literature reveals a more nuanced landscape, indicating overlapping presentations. Several studies have reported a subset of anti-MAG-positive patients with a clinical and electrophysiological profile consistent with CIDP [33^▪▪^,^34^–^37^]. In rare instances, alternative phenotypes such as multifocal neuropathy, small fiber neuropathy, Guillain-Barré-like syndromes, or amyotrophic lateral sclerosis (ALS)-mimics have also been described [34]. Some studies noted that patients with a CIDP-like phenotype despite anti-MAG antibody positivity were less likely to exhibit classical features of anti-MAG neuropathy − such as myelin lamellae widening on biopsy, high antibody titers, or positive immunofluorescence − suggesting potential biological heterogeneity [35,36].

The discriminatory value of anti-MAG antibody titers remains controversial. Although titers are usually higher in typical cases, studies have reported conflicting results. Most laboratories now use the Bühlmann ELISA, yet even high titers (e.g., >10 000 Bühlmann titer units [BTU)] have been found in patients with atypical clinical presentations [34–37]. One recent study identified ≥1500 BTU as an optimal diagnostic threshold (78% sensitivity, 96% specificity), and ≥10 000 BTU as a highly specific threshold (100% specificity) for typical anti-MAG antibody neuropathy, although the control cohort included only 72 CIDP patients [38]. Approximately 5–6% of patients clinically diagnosed with CIDP test positive for anti-MAG antibodies [39,40].

Several studies have also raised concerns that a subset of anti-MAG antibody test results may represent false positives. For instance, anti-MAG antibodies have been documented in patients with IgM monoclonal gammopathy without clinical or electrophysiological evidence of neuropathy, even after prolonged follow-up [41,42]. Moreover, atypical presentations have been reported, including cases resembling Parkinson's disease, ALS, or acute demyelinating neuropathy with respiratory involvement [34,43,44].

Various alternative assays have been evaluated to improve diagnostic accuracy. Antibodies against sulfated glucuronyl paragloboside or human natural killer-1 (HNK-1), the epitope targeted by anti-MAG antibodies, have not consistently increased specificity [45,46]. False-positive results have also been reported with Western blot [41,42,45]. A newly developed cell-based assay targeting HNK-1 has shown promising diagnostic performance but requires further validation [47^▪^].

Distinguishing CIDP from anti-MAG antibody neuropathy carries important prognostic and therapeutic implications. CIDP responds to immunoglobulin, corticosteroids, and plasma exchange, whereas anti-MAG antibody neuropathy generally responds better to anti-CD20 monoclonal antibodies such as rituximab [3^▪^,^33^^▪▪^]. According to the 2021 EAN/PNS criteria, patients with positive anti-MAG antibodies are excluded from a diagnosis of CIDP, which precludes them from receiving CIDP-specific treatments or participating in related clinical trials [3^▪^]. However, some studies suggest that a subset of these patients may benefit from immunoglobulin therapy, despite anti-MAG positivity [48^▪^,^49^].

Future research is needed to clarify the boundaries between these two neuropathies, ideally through the development of more precise diagnostic tools or criteria that guide testing for anti-MAG antibodies only in appropriate clinical contexts.

## CIDP NOT FULFILLING ELECTRODIAGNOSTIC CRITERIA AND THE ROLE OF AXONAL DAMAGE

A notable proportion of patients with suspected CIDP, ranging from 10% to 30% in several studies, do not fulfill established electrodiagnostic criteria, posing significant diagnostic and therapeutic challenges [6,9,18,22,23,24^▪^]. Current guidelines incorporate various supportive investigations, such as elevated CSF protein, nerve imaging, or biopsy, to enhance diagnostic certainty [3^▪^]. However, under the 2010 EFNS/PNS criteria, these ancillary tools were intended to reinforce the diagnosis only in patients who already meet electrodiagnostic criteria for CIDP, and do not permit the diagnosis in those lacking demyelinating features in at least one motor nerve [3^▪^].

Some studies have reported that such patients often undergo limited electrodiagnostic testing, typically involving a relatively small number of nerves [50,51]. This is in line with evidence showing that expanding testing protocols, especially by including additional nerves and proximal segments, can increase the sensitivity of electrodiagnostic criteria [22,23,24^▪^].

Despite not meeting formal criteria, these patients frequently exhibit clinical, demographic, supportive-investigation, and treatment-response profiles comparable to those who do [50,51]. In clinical practice, particularly in tertiary referral centers, these patients may still be considered for immunotherapy, especially when expert evaluation supports the diagnosis [50,51,52^▪^]. In many of these cases, no alternative diagnosis emerges even after long-term follow-up [50,51]. In some patients, repeat electrodiagnostic testing over time reveals demyelinating features that were absent at initial assessment [3^▪^,^51^]. Nonetheless, these cases remain at risk of misdiagnosis, which can have serious consequences, either leading to unnecessary and potentially harmful long-term immunosuppressive treatment, or conversely, depriving patients of effective, disease-modifying therapy. Multiple studies have shown that among patients whose CIDP diagnosis was subsequently revised, most had not fulfilled electrodiagnostic criteria [11,10].

Most experts now agree that patients with suspected CIDP who fail to meet electrodiagnostic criteria should be referred to specialized centers for expert evaluation. The EAN/PNS 2021 guidelines addressed this issue by permitting a diagnosis of possible CIDP in patients with a typical clinical phenotype who do not meet minimal electrodiagnostic criteria, provided they show objective improvement after immunomodulatory treatment and fulfill at least one supportive criterion [3^▪^]. A recent study reported that this modification increased the diagnostic sensitivity of the criteria by only 2–3% [22].

In patients who do not fulfill electrodiagnostic criteria, a comprehensive clinical assessment, careful evaluation of treatment response, and correct application of supportive investigations are essential to support the diagnosis. In this context, recent studies have highlighted the diagnostic value of nerve ultrasound and MRI, which may help identify patients lacking electrodiagnostic evidence of demyelination, reinforcing the complementary role of imaging in the diagnostic evaluation of suspected CIDP [53,54].

Since the early descriptions of CIDP, numerous reports have documented patients without demyelinating abnormalities on nerve conduction studies who exhibited axonal degeneration on nerve biopsy and showed clinical improvement with immunomodulatory therapy, raising the long-standing debate regarding the existence of an ‘axonal CIDP’ variant [55–58]. A recent study by Oh and colleagues described a series of patients with a clinical phenotype resembling CIDP, characterized in most cases by a relapsing–remitting course, proximal weakness, and a clear response to immunomodulatory therapy, in the absence of electrodiagnostic features of demyelination and with nerve biopsies revealing inflammatory infiltrates and prominent axonal degeneration [58]. The authors proposed the term chronic inflammatory axonal polyneuropathy and outlined a set of diagnostic criteria for this condition. Replication in prospective studies is, however, needed − ideally incorporating biomarkers of demyelination (e.g. CSF sphingomyelin [59]) − as it remains possible that limited, nonrepeated electrophysiological assessments and nonrepresentative nerve sampling may have contributed to the absence of demyelinating findings in this study.

Interest in axonal damage in CIDP has increased in recent years, driven by numerous studies highlighting the role of neurofilaments, biomarkers of axonal injury. These studies have shown that neurofilament levels can differentiate CIDP patients from healthy controls, distinguish active disease from remission, decrease following effective treatment, correlate with clinical scores of impairment and disability, and provide prognostic information [60–63,64^▪▪^]. A particularly important and novel implication of these findings is that they challenge the prevailing view of axonal degeneration in CIDP as a late, secondary, and relatively marginal phenomenon, instead suggesting that axonal damage may be an early and dynamic component of disease activity.

## CONCLUSION

Despite advances in diagnostic criteria, the accurate diagnosis of CIDP remains challenging due to persistent controversies regarding electrodiagnostic parameters, disease boundaries with anti-MAG antibody neuropathy, and the recognition of patients who do not meet formal criteria yet respond to treatment. These ambiguities underscore the need for a more flexible, evidence-based diagnostic framework that integrates clinical, electrophysiological, and biomarker data. Future research should focus on refining electrodiagnostic definitions, identifying more precise diagnostic biomarkers, and elucidating the role of axonal damage and its interplay with demyelination and inflammation to enhance diagnostic accuracy and patient stratification.

## Acknowledgements

*None.*

### Financial support and sponsorship

*No external funds were used to perform this study.*

### Conflicts of interest

*Pietro Emiliano Doneddu reports personal fees for Advisory from ArgenX - Belgium and has received travel grants to attend scientific meetings from CSL Behring and Kedrion. Eduardo Nobile-Orazio reports personal fees for Advisory or Scientific Board from ArgenX* − *Belgium, CSL-Behring - Italy and USA, Dianthus* − *USA, Janssen – USA, Kedrion – Italy, LFB – France, Roche – Switzerland, Sanofi – USA, Takeda* − *Italy and USA,. The other authors declare no conflict of interest.*

## REFERENCES AND RECOMMENDED READING

Papers of particular interest, published within the annual period of review, have been highlighted as:

▪ of special interest
▪▪ of outstanding interest
